# Short‐Term Efficacy and Long‐Term Limitations of Self‐Expandable Metallic Stent Placement for Colorectal Obstruction due to Extracolonic Malignancies

**DOI:** 10.1002/deo2.70234

**Published:** 2025-10-31

**Authors:** Masashi Yamamoto, Naoto Osugi, Dai Nakamatsu, Kengo Matsumoto, Koji Fukui, Tsutomu Nishida

**Affiliations:** ^1^ Department of Gastroenterology Toyonaka Municipal Hospital Osaka Japan

**Keywords:** abdominal neoplasms | colonic stent | extracolonic malignancies | intestinal obstruction | self‐expandable metallic stents

## Abstract

**Objectives:**

Although previous studies have investigated colonic stenting for obstruction due to extracolonic malignancies (ECMs), long‐term data—especially concerning quality of life and chemotherapy resumption—remain insufficient.

**Methods:**

Clinical data of 25 patients with ECM‐induced colorectal obstruction were retrospectively analyzed. The primary endpoint was the obstruction‐free duration after stenting. The secondary endpoints included successful stent placement, the clinical course after stent placement, and the outcomes of stent occlusion treatment.

**Results:**

Median age was 69 years, and gastric cancer was the most frequent primary malignancy. Obstruction was caused by peritoneal dissemination (*n* = 21) or direct infiltration (*n* = 4). Stent placement was successful in 86% and 100% of the respective groups, without procedure‐related adverse events. Among 22 successful placements, bowel obstruction relief was achieved in 83.3% with peritoneal dissemination and 100% with direct infiltration. Eleven patients (50%) discontinued intravenous nutrition, and seven (31.8%) resumed chemotherapy. Ten patients (45%) were discharged. The median obstruction‐free duration was 51 days, and the median survival time was 74 days. Two patients with gastric cancer survived over 200 days with resumed chemotherapy. Stent occlusion occurred in three patients; all underwent secondary placement, though salvage surgery was required due to poor clinical outcomes.

**Conclusions:**

Colorectal stenting provides short‐term symptomatic relief in ECM‐induced obstruction; however, long‐term outcomes were limited, likely due to the underlying advanced malignancies. Although secondary stent placement was technically feasible, its effectiveness in recurrent obstruction was poor.

## 1 Introduction

Large bowel obstruction is a serious condition requiring prompt medical intervention. Approximately 82%–93% of cases are attributed to primary colorectal cancer, whereas 1.9%–4.7% result from extracolonic malignancies (ECMs) [1, 2]. Treatment options include tumor resection, bypass surgery, decompression stomas, transanal decompression tubes, and self‐expandable metallic stents (SEMSs). A multidisciplinary approach is essential for selecting the optimal treatment.

Clinical guidelines recommend SEMS placement for colorectal obstruction due to ECMs, based on retrospective evidence showing lower morbidity and mortality than emergent surgery [3, 4, 5, 6, 7, 8, 9]. A prior meta‐analysis found that stent placement for ECM‐induced obstructions was associated with higher rates of technical and clinical failure compared to intraluminal malignancies; however, no significant differences in safety outcomes were observed [10]. While short‐term safety and efficacy are well‐documented, evidence on long‐term quality‐of‐life outcomes, such as cessation of intravenous therapy and discharge to home, remains scarce. Additionally, data on chemotherapy resumption after stent placement are limited, although such patients typically present with advanced, incurable disease.

If the stent becomes occluded, an additional SEMS can be placed, especially if the obstruction is localized, as this is a minimally invasive option. However, data on the outcomes of secondary stent placement for ECM‐related obstructions are scarce. While previous reports revealed that the outcomes of secondary stent placement are poorer than those of initial stent placement, detailed evaluations of these cases and their long‐term implications are lacking [11, 12, 13, 14, 15, 16].

To address these gaps, we conducted a single‐center retrospective analysis of SEMS outcomes for ECM‐related obstructions, focusing on long‐term efficacy and additional stent placement.

## 2 Methods

### 2.1 Study Design

This single‐center retrospective study was conducted at Toyonaka Municipal Hospital. Patients who underwent endoscopic colorectal stent placement for extrinsic colorectal obstruction caused by a malignant tumor originating outside the colorectal wall between January 2009 and March 2023 were included.

Eligibility was determined based on abdominal computed tomography (CT) findings, clinical signs, and laboratory data. Patients with a single‐site obstruction and marked proximal colonic dilation were considered. Exclusion criteria were suspected multiple strictures, gastrointestinal perforation, impending perforation, severe obstructive colitis, long or complex strictures, and lesions near the ileocecal valve or anal verge. For patients meeting these criteria, SEMS placement was selected as the initial treatment due to its minimally invasive nature. Treatment decisions were made collaboratively by gastroenterologists and surgeons.

Patient data, including background information (sex, age, primary malignancies, clinical stage, and colorectal obstruction scoring system [CROSS] score), endoscopic and radiological findings (cause and site of obstruction and stricture length), and the clinical course after SEMS placement (oral intake ability, abdominal symptoms, postoperative CROSS score, need for intravenous nutrition, discharge status, recurrence of obstruction, time to recurrence, follow‐up duration, and survival status), were collected from electronic medical records.

### 2.2 Procedure

Patients received glycerin enemas before the procedure. The colonoscope was advanced to the site of obstruction, and a guidewire was navigated into the proximal colon under fluoroscopy. After the guidewire was inserted, a catheter was inserted along the guidewire, and contrast medium was injected to visualize the stenosis. After a detailed assessment, the endoscopist deployed a metallic stent. Pre‐dilation and argon plasma coagulation (APC) were not performed before initial stent placement. During stent‐in‐stent reintervention, APC was optionally used at the endoscopist's discretion.

For the placement of an Ultraflex uncovered colonic stent (Boston Scientific, Marlborough, MA, USA), the endoscope was withdrawn while the guidewire remained in place, and the stent was advanced along the guidewire and deployed under fluoroscopy. In contrast, for the placement of a WallFlex colonic stent (Boston Scientific, Marlborough, MA, USA), Niti‐S stent (Taewoong Medical Co., Ltd., Gimpo‐si, Gyeonggi‐do, Republic of Korea), ComVi stent (Taewoong Medical Co., Ltd., Gimpo‐si, Gyeonggi‐do, Republic of Korea), or HANAROSTENT Naturfit colon (Boston Scientific, Marlborough, MA, USA), the stent was inserted directly through the endoscope, advanced along the guidewire, and deployed using endoscopic and fluoroscopic guidance.

All initial procedures employed uncovered stents. Ultraflex uncovered stents were used exclusively until 2012, after which WallFlex stents entered use and superseded them. In 2013, Niti‐S stents were introduced, replacing WallFlex stents thereafter. From 2019 onward, Niti‐S stents and HANAROSTENT Naturfits were alternated. For stent‐in‐stent reintervention following occlusion, covered ComVi stents were used.

### 2.3 Definition

The Colorectal Obstruction Scoring System (CROSS), proposed by the Japan Colonic Stent Safe Procedure Research Group, is used to quantify the severity of colonic obstruction on the basis of the need for decompression and oral intake ability.

CROSS 0: Requires continuous decompressive procedures.

CROSS 1: No oral intake.

CROSS 2: Limited to liquid or enteral nutrition.

CROSS 3: Soft solid, low‐residue, or full diet with symptoms of stricture.

CROSS 4: Soft solid, low‐residue, or full diet without stricture symptoms.

Technical success was defined as stent placement at the intended site; major adverse events during the procedure were considered failures. Clinical success was defined as the passage of stool and flatus and the relief of colorectal obstruction, confirmed by X‐ray or CT scan, within 1 week after stent placement. Discontinuation of intravenous nutrition was defined as complete cessation of parenteral support, whether intermittent or continuous. The obstruction‐free duration was defined as the time from the relief of obstruction after stent placement to the recurrence of obstruction.

### 2.4 Outcomes

The primary endpoint was the obstruction‐free duration. The secondary outcomes included technical and clinical success rates, improvements in the CROSS score, adverse events, ability to discontinue intravenous nutrition, chemotherapy resumption, likelihood of discharge to home care, and clinical outcomes of additional stent placement.

### 2.5 Statistical Analysis

Continuous variables are expressed as medians with interquartile ranges, and categorical variables are presented as frequencies (%). Categorical associations were tested using Fisher's exact test. Univariate logistic regression estimated odds ratios and 95% confidence intervals for predictors of long‐term outcomes, including discontinuation of intravenous nutrition and discharge to home. Changes in CROSS scores were analyzed using the Wilcoxon signed‐rank sum test, and obstruction‐free duration was analyzed using the Kaplan–Meier method. Statistical significance was defined as *p* < 0.05. Analyses utilized JMP (version 16.20; SAS Institute Inc.).

## 3 Results

Twenty‐five patients underwent initial SEMS placement for ECM‐related obstruction. The median age of the patients was 69 years, and 52% (13 patients) were male. Gastric cancer was the most common primary malignancy, constituting 44% (11 patients) of all cases, followed by recurrent colorectal cancer, pancreatic cancer, gallbladder cancer, and other conditions. Twenty‐four of 25 patients (96%) were diagnosed with stage IV disease, while the remaining patient, with stage IIB disease, had unresectable pancreatic cancer. None of the patients qualified for curative surgical resection at the time of diagnosis of colorectal obstruction. The median time from the diagnosis of the primary malignancy to the onset of colorectal obstruction was 722 days. Peritoneal dissemination was identified as the cause of obstruction in 84% of patients (21 patients), whereas direct invasion from adjacent organ masses was the cause of obstruction in the remaining 16% (four patients). The median CROSS score was one at the time of stent placement (Table 1). All SEMS placement procedures were performed for palliative purposes, and none served as a bridge to surgical intervention.

**TABLE 1:** Patient characteristics with large bowel obstruction caused by extracolonic malignancy (ECM).

	*n* = 25
Sex, Male, *n* (%)	13 (52%)
Age (year), median (IQR)	69 (54.5, 82)
Primary malignancies, *n* (%)	
Gastric cancer	11 (44%)
Postoperative colorectal cancer dissemination	4 (16%)
Pancreatic cancer	3 (12%)
Gallbladder cancer	3 (12%)
Others	4 (16%)
Clinical Stage, Stage IV, *n* (%)	24 (96%)
Time from diagnosis to obstruction, days, median (IQR)	722 (166, 1078)
Cause of Colorectal Obstruction	
Peri‐colorectal tumor dissemination	21 (84%)
Direct tumor invasion from the surrounding organ	4 (16%)
Aim of stent deployment	
Palliative setting	25 (100%)
Bridge to surgery	0 (0%)
Pre‐procedural CROSS, median (IQR)	1 (0‐3)

*IQR: interquartile range.

The median stricture length was 4 cm. In 24 cases (96%), strictures were located between the rectum and transverse colon. All the stents used were uncovered and had a median length of 9.5 cm. The technical success rate was 88%; in the remaining three cases, severe curvature of the lumen caused by peritoneal dissemination prevented guidewire insertion, making stent placement unfeasible. Furthermore, no perforation, bleeding events, or stent migration were observed among the 25 patients (Table 2).

**TABLE 2:** Procedural outcomes of metallic stent deployment.

	*n* = 25
Site of colorectal obstruction	
Ascending colon	1 (4%)
Transverse colon	7 (28%)
Descending colon	6 (24%)
Sigmoid colon	6 (24%)
Rectum	5 (20%)
Length of stenosis (cm), median (IQR)	4 (3.2, 5.0)
Procedure time (minutes), median (IQR)	28 (20, 48)
Technical success, *n* (%)	22 (88%)
Deployed stent type, uncovered, *n* (%)	22 (100%)
Ultraflex uncovered colonic stent	2
WallFlex colonic stent	3
Niti‐S stent	12
HANAROSTENT Naturfit colon	5
Deployed stent length (cm), median (IQR)	9.5 (8, 12)
Adverse events	
Bleeding	0
Perforation	0
Stent migration	0
Others	0

*IQR: interquartile range.

**FIGURE 1:**
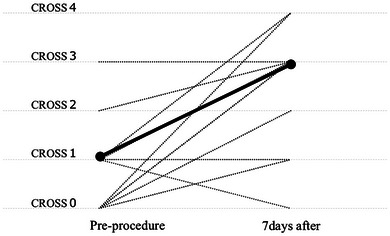
Changes in the colorectal obstruction scoring system (CROSS) score before and seven days after successful stent placement. The dotted lines illustrate individual changes in CROSS scores for each patient, whereas the bold solid line indicates the median change across all patients (*p* < 0.01).

**FIGURE 2:**
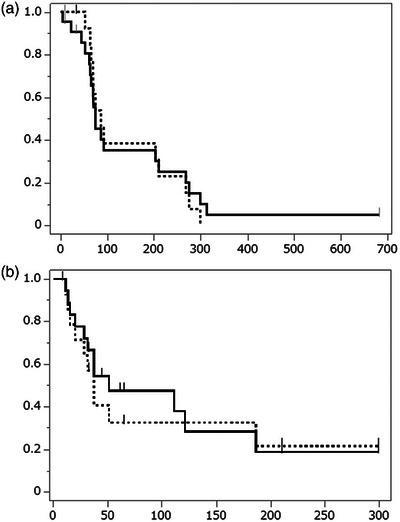
(a) Kaplan‒Meier curves depicting survival time following stent placement. The horizontal axis represents the survival period (in days), whereas the vertical axis represents the survival probability. The solid line represents the survival duration for the entire cohort of patients, with a median of 74 days (interquartile range, 63–268 days). The dotted line indicates the survival duration for patients in the peritoneal dissemination group who experienced relief of intestinal obstruction, with a median of 86 days (interquartile range: 69–210 days). (b) Kaplan‒Meier curves depicting the obstruction‐free duration in patients with successful obstruction relief after stenting (time until recurrence of intestinal obstruction). The horizontal axis represents the obstruction‐free duration (in days), whereas the vertical axis represents the obstruction‐free rate. The solid line indicates the obstruction‐free duration for the entire cohort of patients, with a median of 51 days (interquartile range: 28–186 days). The dotted line represents the obstruction‐free duration for the disseminated case group, with a median of 37 days (interquartile range, 20–186 days).

Nineteen patients showed clinical and radiographic improvements in intestinal obstruction within a week after the procedure. The clinical success rate across all cases was 76%. Median CROSS score significantly increased from 1 to 3 (*p* < 0.0001; Figure 1). Among 22 technically successful cases, 15 out of 18 patients (83.3%) with peritoneal dissemination achieved clinical success, while the patients with direct invasion demonstrated a 100% clinical success rate. Longer‐term outcomes included: sixteen patients (72.7%) experienced prolonged abdominal pain and bloating due to their advanced malignancies. In terms of oral intake, 17 patients (77%) were able to resume eating; however, only 11 patients (50%) received sufficient nutrition to discontinue intravenous nutrition. Seven patients (31.8%) resumed chemotherapy following stent placement. Only 10 patients (45%) were ultimately discharged home, while the remainder either died in the hospital or were transferred to hospice care. No difference in clinical outcomes was identified across stent types. The median follow‐up duration was 77 days (Table 3). Kaplan‐Meier analysis revealed a median survival of 74 days after stent placement. In the peritoneal dissemination group, even when the colorectal obstruction was successfully resolved, the median survival remained short, at 86 days (Figure 2a). To explore factors associated with long‐term outcomes, including discontinuation of intravenous nutrition and discharge to home, we performed univariate analyses; however, no statistically significant predictors were identified (Tables 4 and 5).

**TABLE 3:** Clinical course after successful stent deployment.

	*n* = 22
Follow‐up period (days), median (IQR)	77 (56.8, 224.5)
Improvement of colorectal obstruction, *n* (%)	19 (86.4%)
Relief of abdominal pain and distension, *n* (%)	6 (27.3%)
Resumption of oral food intake, *n* (%)	17 (77.3%)
Discontinuation of intravenous nutrition, *n* (%)	11 (50%)
Discharge to home, *n* (%)	10 (45%)
Post‐Stent chemotherapy, *n* (%)	7 (31.8%)
Relapse of bowel obstruction, *n* (%)	12 (63.2%)
Secondary stent insertion	3
Duration to relapse of obstruction (days), median (IQR)	34 (16, 96)
Peri‐colorectal dissemination	30 (11, 41)
Direct tumor invasion	116 (111, 121)

*IQR: interquartile range.

**TABLE 4:** Univariate analysis of factors contributing to discontinuation of intravenous nutrition.

	Univariate logistic analysis		
Variables	Odds ratio	95% CI	*p*‐Value
Sex, Male, yes	1.44	0.13–3.72	1.0000
Age, 69 and over, yes	4.67	0.04–1.31	0.1984
Stenosis location, distal colon, yes	1.52	0.25–9.29	1.0000
Stenosis length, 4 cm and over, yes	0.48	0.38–11.6	0.6699
Cause of obstruction, dissemination, yes	1.00	0.11–8.73	1.0000
Primary malignancy, gastric cancer, yes	4.67	0.77–28.5	0.1984
Time from diagnosis to obstruction, 722 days or longer, yes	7.11	0.14–0.92	0.0861

Abbreviation: CI: confidence interval.

**TABLE 5:** Univariate analysis of factors contributing to discharge to home.

	Univariate logistic analysis		
Variables	Odds ratio	95% CI	*p*‐Value
Sex, Male, yes	2.10	0.09–2.63	0.6699
Age, 69 and over, yes	0.33	0.06–1.91	0.3913
Stenosis location, distal colon, yes	1.17	0.19–7.12	1.0000
Stenosis length, 4 cm and over, yes	0.71	0.26–7.58	1.0000
Cause of obstruction, dissemination, yes	0.80	0.09–7.00	1.0000
Primary malignancy, gastric cancer, yes	3.00	0.52–17.2	0.3913
Time from diagnosis to obstruction, 722 days or longer, yes	4.67	0.04–1.31	0.1984

Abbreviation: CI: confidence interval.

Intestinal obstruction recurred in 12 of 19 patients (63.2%) who initially experienced improvement, and the median time to recurrence was 34 days (30 days in the peritoneal dissemination group; 116 days in the direct invasion group; Table 3). Kaplan‐Meier analysis of 19 patients with obstruction relief showed an overall median obstruction‐free duration of 51 days and a median obstruction‐free duration of 37 days in the peritoneal dissemination group (Figure 2b).

**FIGURE 3:**
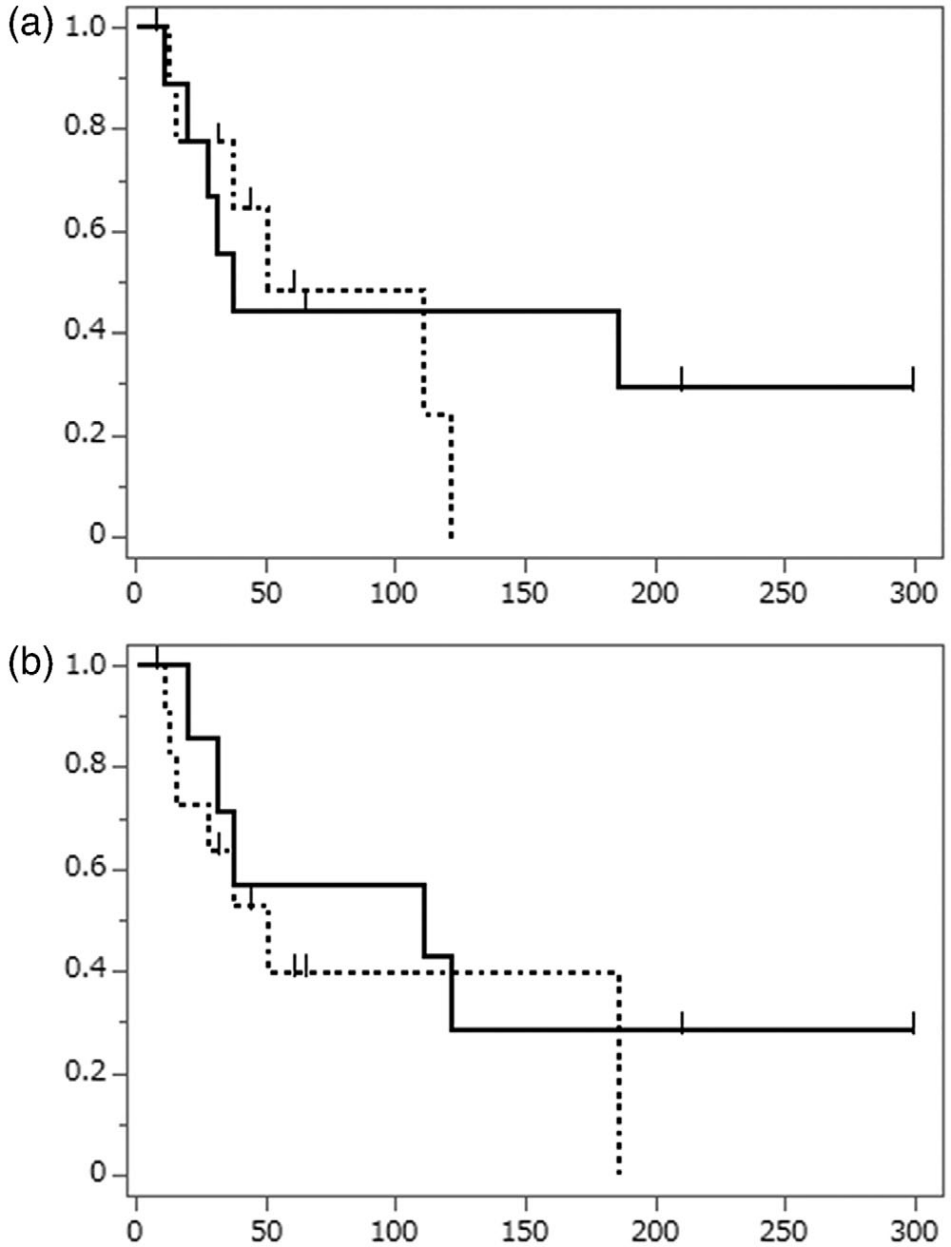
(a) Kaplan‐Meier curves illustrating the obstruction‐free duration in patients with successful obstruction relief after stenting (time until recurrence of intestinal obstruction). The horizontal axis represents the obstruction‐free duration (in days), while the vertical axis represents the obstruction‐free rate. The solid line corresponds to the gastric cancer group, which presented a median of 37 obstruction‐free days (interquartile range not calculable due to the limited number of patients). The dotted line represents the non‐gastric cancer group, who presented a median of 51 obstruction‐free days (interquartile range: 37–111 days). (b) Kaplan‐Meier curves illustrating the obstruction‐free duration in patients with successful obstruction relief after stenting (time until recurrence of intestinal obstruction). The horizontal axis represents the obstruction‐free duration (in days), while the vertical axis represents the obstruction‐free rate. The solid line represents patients who underwent subsequent chemotherapy, presenting a median of 111 obstruction‐free days (interquartile range not calculable due to the limited number of patients). The dotted line depicts patients who did not undergo subsequent chemotherapy, with a median of 51 obstruction‐free days (interquartile range: 15–186 days).

Among the seven patients without documented recurrence, five died or transitioned to hospice care within a short period (median follow‐up: 44 days). On the other hand, the remaining two—both with gastric cancer and peritoneal dissemination—resumed chemotherapy after stent placement and survived over 200 days without recurrence. Kaplan‐Meier analysis revealed that the median obstruction‐free duration was 37 days for patients with gastric cancer and 51 days for patients with nongastric cancer (Figure 3a). Notably, patients who underwent chemotherapy after stent placement had a longer median obstruction‐free duration (111 days) compared with those who did not receive chemotherapy (51 days) (Figure 3b).

Of the 12 patients with recurrent intestinal obstruction, nine had multiple strictures, making additional stent placement and surgical intervention unfeasible. In the remaining three patients, the lumen of the initial stent was occluded, causing obstruction solely at the site of the initial stent. Endoscopic and histopathologic findings in all three patients revealed no malignancy at the obstruction site, instead suggesting hyperplasia of otherwise normal mucosa. Additional SEMSs were placed because of the minimal invasiveness of the procedure. The additional stents that were placed were covered stents.

**FIGURE 4:**
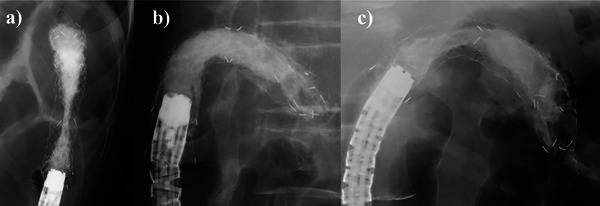
Images of additional stent placement after contrast agent injection. (a) Obstruction of the descending colon in a patient with metastatic gastric cancer. Despite additional stent placement, significant hyperplasia prevented full stent expansion. (b) Obstruction of the descending colon due to direct invasion by pancreatic tail cancer. A stent was inserted after argon plasma coagulation (APC) ablation and fully expanded; however, it migrated on the ninth day. (c) Obstruction of the descending colon due to direct invasion by pancreatic tail cancer. An additional stent was placed without APC due to mild hyperplasia; however, obstructions caused by fecal masses remained frequent, leading to the need for bypass surgery.

Patient 1: A 47‐year‐old female with descending colon obstruction due to gastric cancer‐related peritoneal dissemination. Reobstruction was observed 31 days after the initial stent placement procedure. An additional stent was placed (Figure 4a), but the obstruction persisted, so an ileal‐sigmoid colon bypass was needed. The patient continued chemotherapy and survived for 229 days after the procedure.

Patient 2: A 76‐year‐old female presented with descending colon obstruction due to direct invasion of the pancreatic tail. Reobstruction was observed 111 days after the initial stent placement procedure. APC was performed to reduce hyperplasia, followed by additional SEMS placement (Figure 4b). However, nine days later, both stents had migrated, resulting in reobstruction. Surgical resection of the pancreatic tail and left‐sided colon was performed, allowing chemotherapy to be continued. She survived for 560 days.

Patient 3: A 50‐year‐old male with descending colon obstruction caused by pancreatic tail cancer invasion. Reobstruction was observed 121 days after the initial stent placement procedure, and an additional stent was placed without APC (Figure 4c). The first episode of fecal impaction occurred 10 days after the second stent placement, marking the onset of repeated episodes of fecal impaction. These recurrent obstructions limited adequate chemotherapy administration and ultimately required treatment with transverse colon‐sigmoid colon bypass. The patient continued chemotherapy and survived for 181 days after the procedure.

Despite the placement of additional stents, the obstructions were not completely resolved in any of the three patients, so surgical intervention was needed.

**FIGURE 5:**
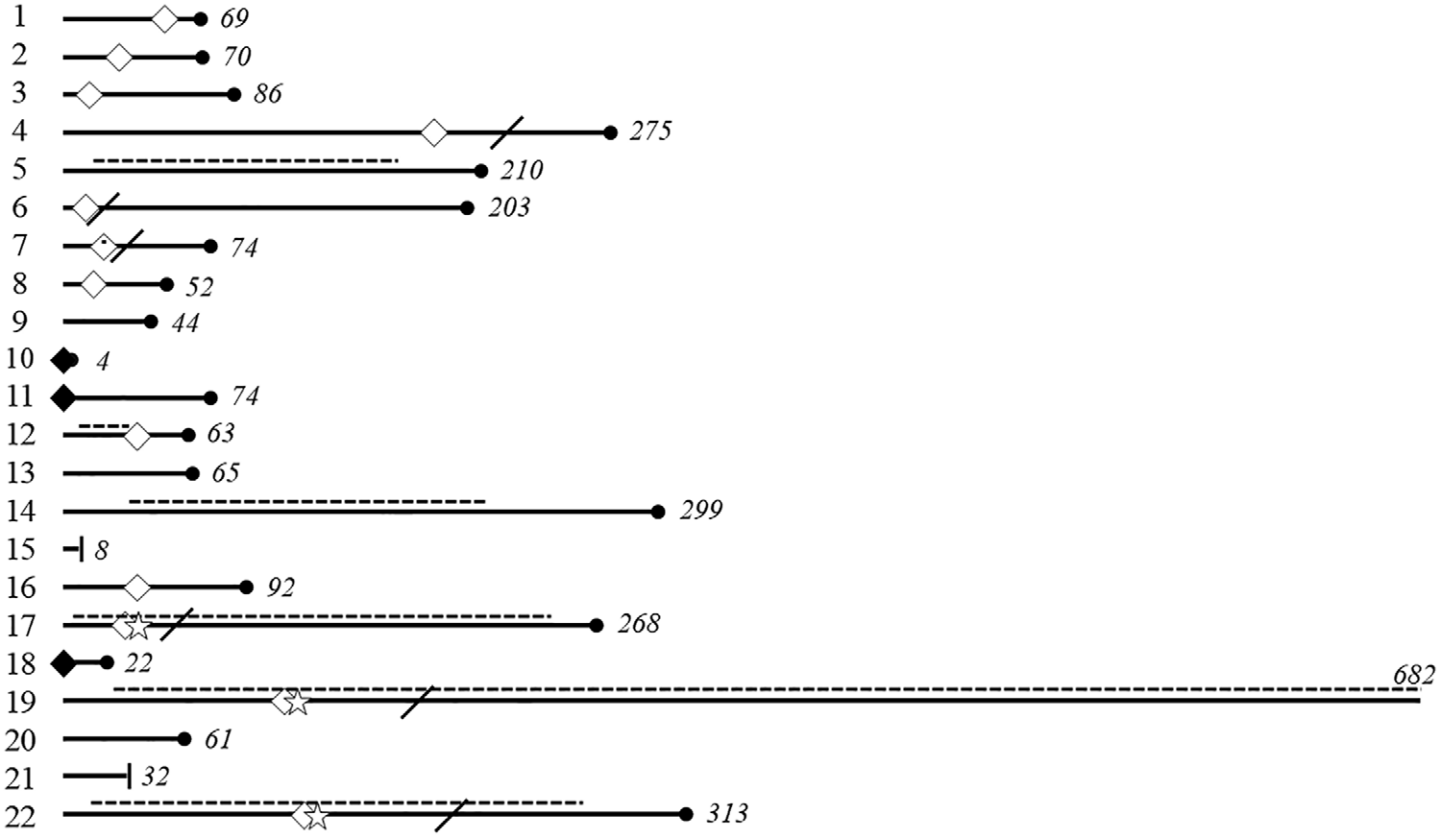
Post‐stenting clinical course of 22 patients with successful initial self‐expandable metallic stent (SEMS) placement. Each row represents an individual case with successful initial SEMS placement. Solid horizontal bars indicate the follow‐up duration after initial stent placement, with the number of days shown at the right of each bar. A dotted line above each bar indicates the duration of chemotherapy administration. A black diamond symbol denotes unresolved obstruction persisting beyond 1 week after initial stent placement. A white diamond symbol marks obstruction recurrence after initial improvement. A star symbol indicates additional SEMS placement after obstruction recurrence. A diagonal line represents additional surgery after obstruction recurrence. A black dot at the right end indicates confirmed death, while a vertical tick mark denotes censoring due to the end of follow‐up in surviving cases.

Figure 5 outlines the clinical course of 22 patients with successful SEMS placement, including chemotherapy, obstruction recurrence, additional stenting, and surgery.

## 4 Discussion

Our study revealed favorable short‐term outcomes of SEMS placement in patients with large bowel obstruction caused by ECM. The technical success rate was 88%. Clinical success was achieved in over 80% of patients with peritoneal dissemination, and notably, in all cases with direct infiltration. These results align with those of previous studies, which revealed success rates between 54.5% and 91.1% for improving intestinal obstruction [5, 6, 7, 8, 17]. In a study by Shin, the rate of SEMS migration used in patients with ECM‐related colorectal obstruction was 2.6% for uncovered stents and 21.1% for covered stents [18]. On the basis of these findings, we decided to use only uncovered stents and observed no instances of stent migration. Furthermore, no adverse events, such as bleeding or perforation, were recorded, highlighting the safety of this approach. Our findings support SEMS as a minimally invasive option for ECM‐related obstructions in advanced malignancies, in line with current guidelines.

However, long‐term outcomes of SEMS for ECM‐related colorectal obstruction remain suboptimal. This study, to our knowledge, is the first to systematically assess underreported palliative indicators, including intravenous nutrition discontinuation and discharge rates. Despite radiologically confirmed improvement in obstruction, more than 70% of patients continue to experience symptoms such as abdominal pain, bloating, and poor appetite. As a result, only 50% of patients were able to discontinue intravenous nutrition, and only 45% were discharged home. These findings contrast with the more favorable discharge rates reported for palliative stent placement in patients with colorectal cancer [19]. The recurrence of intestinal obstruction was a significant issue, affecting 63.2% of patients, with a median time to recurrence of 30 days in patients with peritoneal dissemination. Although SEMS placement is a promising solution for acute obstruction, its long‐term efficacy, particularly in cases with dissemination, remains limited. Importantly, these unfavorable outcomes may not solely reflect the inherent limitations of SEMS placement but rather the advanced progression of the underlying disease, such as peritoneal carcinomatosis or multiple metastases. This underscores the need for further studies to refine treatment strategies and improve long‐term symptom management in this patient population.

Moreover, few studies have reported the feasibility and clinical course of chemotherapy following stent placement for ECM‐induced obstruction. In our cohort, 50% of patients were able to resume chemotherapy post‐stenting. Notably, two patients with gastric cancer and peritoneal dissemination who resumed chemotherapy achieved prolonged survival, remaining obstruction‐free for well over 200 days. Although Kaplan–Meier analysis revealed no significant differences, patients who resumed chemotherapy tended to have longer patency. Recent advances in combination chemotherapy, molecular targeted agents, and immune checkpoint inhibitors have improved outcomes for inoperable advanced cancers [20, 21]. When patients maintain good performance status and qualify for pharmacotherapy post‐stenting, long‐term efficacy may be achievable—even with peritoneal dissemination.

Three patients in our study required additional SEMS placement to resolve the mucosal hyperplasia‐induced occlusion in the initial stent. Although these procedures were technically successful and adverse event‐free, all three patients experienced stent expansion failure or very early stent migration and therefore required surgical intervention. The outcomes of additional stent placement were evaluated in only a few studies, with previous studies revealing that poor results were linked to factors such as short initial stent patency, male sex, and right‐sided colon obstructions [11, 12, 13, 14, 15, 16]. In our study, all reobstructions occurred in the left‐sided colon within 1–4 months after the initial stent placement procedure. Stents placed without additional APC ablation did not expand well, whereas those placed with APC treatment exhibited 60%–90% lumen patency and were at risk for early migration. Further research should explore the role of lighter APC ablation in optimizing the outcomes of additional SEMS placement.

This study had several limitations. First, the sample size was small, which may have limited the generalizability of the findings. Second, the retrospective, single‐center design could have introduced bias and restricted the scope of the results. Despite these limitations, the findings of this study provided valuable insights into the challenges and outcomes of SEMS placement for ECM‐related colorectal obstructions and highlighted areas for future research.

In conclusion, SEMS placement appears to be an effective and minimally invasive option for managing acute colorectal obstruction caused by ECM, particularly in emergency settings. Short‐term outcomes, including technical and clinical success rates, were favorable; however, long‐term efficacy was limited by the advanced progression of the underlying disease, resulting in persistent symptoms, the inability to discontinue intravenous fluid supplementation, and difficulty in discharging home. Furthermore, chemotherapy could not be resumed in many cases. Re‐stenting for stent occlusion provided only marginal benefits. Given the limited sample size of this study, further research is warranted to enhance scientific rigor and explore optimal strategies for long‐term symptom control.

## Author Contributions

All the authors helped design this single‐center retrospective study and collect case data from medical records. Data were collected and then analyzed by all the authors during discussions at the conference. All the authors reviewed and commented on the manuscript. All the authors have approved this submission and assume full responsibility for the final manuscript.

## Conflicts of Interest

The authors declare no conflicts of interest.

## Funding

No specific funding was received for the study.

## Ethics Statement

This study was approved by the Institutional Review Board of Toyonaka Municipal Hospital (No. 2024‐12‐01). This study was conducted in accordance with the principles of the Declaration of Helsinki.

## Consent

Patient consent was obtained using the opt‐out method on the hospital website.

## Clinical Trial Registration

N/A.
